# Supplementary material for: The Impact of Working from Home on Mental Health: A Cross-Sectional Study of Canadian Worker’s Mental Health during the Third Wave of the COVID-19 Pandemic
Source: Int J Environ Res Public Health. 2022 Sep 14;19(18):11588. doi: 10.3390/ijerph191811588 (PMC9517068; doi:10.3390/ijerph191811588)
Supplement: Supplementary file 1 [file ijerph-19-11588-s001.zip › ijerph-1877037-supplementary (1).pdf]

Supplementary File S1. Independent Variables Included in Initial Multivariable Binary Regression Model

**Variable**	**Description**	**Response Level**
work_from_home	During the COVID-19 pandemic, how often have you worked from home?	8888:Not Working During COVID
		All of the time
		Most of the time
		Not at all
		Some of the time
		Very little of the time
Income	What is your best estimate of your total household income received by all household members, from all sources, before taxes and deductions, during the year ending December 31, 2020? Note: Income can come from various sources such as from work, investments, pensions or government. Examples include Employment Insurance, Social Assistance, Child Tax Benefit and other income such as child support, spousal support (alimony) and rental income.	$10,000 to $14,999
		$100,000 to $149,999
		$15,000 to $19,999
		$150,000 to $199,999
		$20,000 to $24,999
		$200,000 or more
		$25,000 to $29,999
		$30,000 to $34,999
		$35,000 to $39,999
		$40,000 to $44,999
		$45,000 to $49,999
		$5,000 to $9,999
		$50,000 to $59,999
		$60,000 to $69,999
		$70,000 to $79,999
		$80,000 to $89,999
		$90,000 to $99,999
		Under $5,000
gender	How do you identify, in terms of gender? - Selected Choice	Man
		Non-binary
		Woman
ethnicity	Which of the following best describes your race or ethnicity? - Selected Choice	African, Caribbean, or Black
		Arab
		Chinese
		Filipino
		Indigenous

		Japanese
		Korean
		Latin American
		None of the above, I identify as
		South Asian (e.g., East Indian, Pakistani, Sri Lankan, etc.)
		Southeast Asian (e.g., Vietnamese, Cambodian, Laotian, Thai, etc.)
		West Asian (e.g., Iranian, Afghan, etc.)
		White
identity_newcomers	Do you belong to any of the following groups? (Check all that apply) - Newcomers (e.g., Recent immigrants and refugees, i.e. being in Canada for less than 10 years)	8888: Not Selected
		Newcomers (e.g., Recent immigrants and refugees, i.e. being in Canada for less than 10 years)
age	How old are you (in years)?	--
educational_attainment	Derrived Variable: Educational Attainment	Apprenticeship or trades certificate or diploma
		Bachelor's degree
		College, CEGEP or other non-university certificate or diploma
		Degree in medicine, dentistry, veterinary medicine or optometry
		Earned doctorate
		High School Diploma or Lower
		Master's degree
		University certificate or diploma above bachelor level
		University certificate or diploma below bachelor level
money_never_have	How well do each of the statements describe you? - Because of my money situation, I feel like I will never have the things I want in life.	Completely
		Not at all
		Somewhat
		Very Little
		Very Well

money_concerned	How well do each of the statements describe you? - I am concerned that the money I have or will save won t last.	Completely<br>Not at all<br>Somewhat<br>Very Little<br>Very Well
money_finances_control	How frequent are each of the statements below true for you? - My finances control my life.	Always<br>Never<br>Often<br>Rarely<br>Sometimes
work_stress	How frequent are each of the statements below true for you? - I feel stress about my job even when I am not at work.	8888:Not Working During COVID<br>Always<br>Never<br>Often<br>Rarely<br>Sometimes
work_unsustainable	How well do each of the statements describe you? - I feel that my work-load is unsustainable.	8888:Not Working During COVID<br>Completely<br>Not at all<br>Somewhat<br>Very Little<br>Very Well
work_control	How well do each of the statements describe you? - I have a lot of control over how I do my work.	8888:Not Working During COVID<br>Completely<br>Not at all<br>Somewhat<br>Very Little<br>Very Well
time_working	Please rate whether you spend too much time, too little time, or just the right amount of time doing each of the following activities: - working	Just the right amount<br>Too little<br>Too much
burnout_helpless	When you think about your life overall, how	Almost never<br>Always<br>Never

	often do you feel the following? - Helpless	Often
		Rarely
		Sometimes
		Very Often
employment_impact_laid_off	Did COVID-19 impact your employment in any of the following ways? (Check all that apply) - I was laid off temporarily or permanently	8888: Not Selected
		I was laid off temporarily or permanently
employment_impact_not_employed	Did COVID-19 impact your employment in any of the following ways? (Check all that apply) - I was not employed or self-employed before the COVID-19	8888: Not Selected
		I was not employed or self-employed before the COVID-19
employment_impact_increased_hours	Did COVID-19 impact your employment in any of the following ways? (Check all that apply) - I had my hours of work increased	8888: Not Selected
		I had my hours of work increased
employment_impact_decreased_hours	Did COVID-19 impact your employment in any of the following ways? (Check all that apply) - I had my hours of work reduced	8888: Not Selected
		I had my hours of work reduced
employment_impact_new_job	Did COVID-19 impact your employment in any of the following ways? (Check all that	8888: Not Selected
		I got a new job

	apply) - I got a new job	
employment_impact_no_change	Did COVID-19 impact your employment in any of the following ways? (Check all that apply) - I have had no change in my hours of work	8888: Not Selected
		I have had no change in my hours of work
employment	Derrived Variable: Employment (based on hours worked)	Full Time
		No Work
		Overtime
		Part Time
occupation	Please use the drop-down menu below to identify the best matching description of your occupation:Note: If you are unemployed due to the pandemic, please indicate the job that you were doing before you became unemploymed.	Business, finance and administration occupations
		Health occupations
		Management occupations
		Natural and applied sciences and related occupations
		Natural resources, agriculture and related production occupations
		Occupations in art, culture, recreation and sport
		Occupations in education, law and social, community and government services
		Occupations in manufacturing and utilities
		Sales and service occupations
		Trades, transport and equipment operators and related occupations
work_dignity	How well do each of the statements describe you? - I feel that I am treated with dignity and respect in my workplace.	8888:Not Working During COVID
		Completely
		Not at all
		Somewhat
		Very Little
		Very Well
housing_cost	How much do you pay for housing each month? Note: Please Include	--
		Other
		Own it outright (including condos)
		Own it with mortgage or loan (including condos)

	rent or mortgage payments, strata fees, insurance payments, and, if applicable, property taxes.	Part own and part rent (shared ownership such as co-op)
		Rent at no costs, i.e. rent-free in relativeís/friendís property\
		Rent it with subsidies, i.e. you pay a below-market rate (including rent geared to income, social housing, government assisted housing, housing allowances, etc.)
		Rent it without subsidies, i.e. you pay a market rate
housing_satisfaction	How satisfied or dissatisfied are you with your current living arrangements?	Dissatisfied
		Satisfied
		Very Dissatisfied
		Very Satisfied
commute_time	How many minutes does it usually take you to get from home to work or school?	0 minutes, I work from home
		15 to 29 minutes
		30 to 44 minutes
		45 to 59 minutes
		60 minutes and over
		8888: Only Worked From Home During COVID
		8888:Not Working During COVID
		Less than 15 minutes
commute_mode	How do you usually get to school or work?	8888: Only Worked From Home During COVID
		8888:Not Working During COVID
		Bicycle
		Bus
		Car, truck or van - as a driver
		Car, truck or van - as a passenger
		Light rail, streetcar or commuter train
		Motorcycle, scooter or moped
		Other method
		Passenger ferry
		Subway or elevated rail
		Walked to work
covid_masks	To what extent are you currently following the COVID-19 prevention practices listed below? - Wear a mask in public	Not at all
		Somewhat
		Very Closely

covid_distancing	To what extent are you currently following the COVID-19 prevention practices listed below? - Physically distance yourself by 2 metres from others	Not at all
		Somewhat
		Very Closely
vaccinated	Have you received a COVID-19 Vaccine?	No
		Yes, one dose
		Yes, two doses
self_rated_mental_health	At the present time, would you say your MENTAL HEALTH is:	Excellent
		Very good
		Good
		Fair
		Poor
